# Electrodermal Activity Sensor for Classification of Calm/Distress Condition

**DOI:** 10.3390/s17102324

**Published:** 2017-10-12

**Authors:** Roberto Zangróniz, Arturo Martínez-Rodrigo, José Manuel Pastor, María T. López, Antonio Fernández-Caballero

**Affiliations:** 1 Instituto de Tecnologías Audiovisuales, Universidad de Castilla-La Mancha, 16071 Cuenca, Spain; arturo.martinez@uclm.es (A.M.-R.); josemanuel.pastor@uclm.es (J.M.P.); 2 Instituto de Investigación en Informática, Universidad de Castilla-La Mancha, 02071 Albacete, Spain; Maria.LBonal@uclm.es (M.T.L.); Antonio.Fdez@uclm.es (A.F.-C.)

**Keywords:** wearable, electrodermal activity, arousal, valence, distress, calmness

## Abstract

This article introduces a new and unobtrusive wearable monitoring device based on electrodermal activity (EDA) to be used in health-related computing systems. This paper introduces the description of the wearable device capable of acquiring the EDA of a subject in order to detect his/her calm/distress condition from the acquired physiological signals. The lightweight wearable device is placed in the wrist of the subject to allow continuous physiological measurements. With the aim of validating the correct operation of the wearable EDA device, pictures from the International Affective Picture System are used in a control experiment involving fifty participants. The collected signals are processed, features are extracted and a statistical analysis is performed on the calm/distress condition classification. The results show that the wearable device solely based on EDA signal processing reports around 89% accuracy when distinguishing calm condition from distress condition.

## 1. Introduction

Early mental stress detection can prevent many health problems related to distress (negative stress). Therefore, there is an urgent need to create new technologies to monitor the physical and mental health of people during their daily life. Fortunately, some efforts are being carried out towards monitoring and regulating people’s arousal state [1,2,3], which is indicative of stress or mental illness [4,5,6,7]. Thus, the lack of human–machine interfaces in interpreting the subjects’ emotional states is being faced with the important aim of understanding and managing personal well-being regarding mental health state.

In this regard, a number of physiological features have been widely used in the literature [8,9]. These features use to measure alterations in the central nervous system [10,11,12]. One of these physiological markers corresponds to electrodermal activity (EDA). The utilization of EDA is excellent in assessing the arousal level, as it is able to quantify changes in the sympathetic nervous system. In order to continuously measure the EDA signal from the subjects, wearable sensors are the most appropriate in real mobility situations, given their performance in providing detailed user-specific information. Moreover, wearable sensors are greatly valued due to their light weight and their wireless communication capacities with either a computer or other wearable sensors [13].

In this sense, there are several low-cost solutions for wearable long-term EDA monitoring. For instance, the similarity of signals between a prototype of the wearable Moodmetric EDA Ring is compared with a laboratory-grade skin conductance sensor in a psycho-physiological experiment [14]. Recently, a pilot study of EDA measurements conducted during a trade fair has been presented [15].

This paper describes and assesses the performance of a new wearable electrodermal activity-based device in classifying distress or calm conditions. Section 2 introduces the hardware description of the wearable. Section 3 introduces an experiment that has been designed in order to assess the validity of the proposed wearable. The experimental design and the description of the study population are presented in Section 3.1 and Section 3.2, respectively. Then, in Section 4, the segmentation and processing of the several signals (Section 4.1), as well as the feature extraction process (see Section 4.2) and the statistical analysis of the classification capabilities (see Section 4.3), are described in extensive. Afterwards, the results are offered in Section 5, and Section 6 includes the most outstanding discussion and conclusions related to this work.

## 2. Signal Monitoring and Hardware Description

The electrodermal activity (EDA) measures the changes in conductivity produced in the skin due to increases in the activity of sweat glands. The preferred sites for EDA measures are located in the palms of the hands and the soles of the feet. The eccrine glands secrete sweat due to external stimuli and endogenous processes (memory, attention, vigilance, motor commitment, etc.), filling the skin pores and increasing the conductivity [16]. The sudomotor nerve activity (SMNA) is responsible for triggering the sudomotor fibers that activate the sweat glands. SMNA controls different physiological processes, like thermal regulation or sensory discrimination [17,18]. Nevertheless, it has been reported that SMNA is linked to the emotional state, particularly influencing the arousal dimension [19].

In the scientific literature two different methodologies are described for measuring EDA signals. On the one hand, the endosomatic methodology (ESM) is characterized by not using external current to acquire the EDA signals. Although this method is non-intrusive, it is difficult to interpret the recorded signals [19]. This is the main reason why the exosomatic methodology (EXM) is commonly used to measure EDA signals. EXM recording is performed by using direct current (DC-EXM), or alternating current (AC-EXM). Notice that most EDA studies have been done with DC-EXM, because the empirical superiority of the AC-EXM variant has not been demonstrated [19]. Consequently, this work has performed a DC-EXM methodology with a constant current source.

EDA signals are composed by the superposition of two different components. On the one hand, the phasic component or skin conductance response (SCR) can be observed when the sudomotor nerve is activated. Given this relationship, SCR has been widely used to measure the sympathetic nervous system [8,20,21]. From a morphological point of view, SCR is represented by a peak or a burst of peaks with different amplitudes, slopes and decays depending on the nature of the stimulus [19]. On the other hand, the tonic component, or skin conductance level (SCL), represents the base line of the skin conductance. SCL varies among people, depending on their physiological states and autonomic regulation [22]. Thus, the EDA morphology is represented by a fast changing SCR signal modulated by a slowly varying SCL component. Given the slow response of the SCL component, the useful information ranges from 0 to 0.05 Hz. Similarly, the energy of the SCR component ranges from 0.05 to 1.5 Hz. Indeed, it has been reported that the average activation rate of sudomotor fibers (responsible of the SCR component) are measured at 0.62 Hz [23].

**Figure 1:**
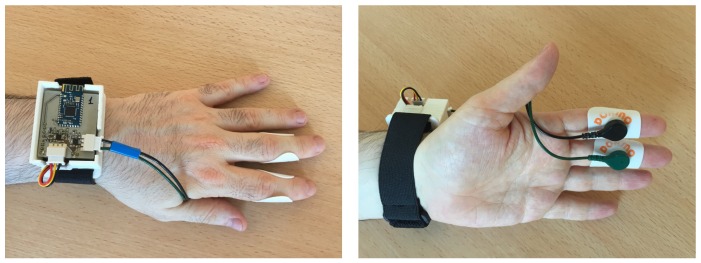
Prototype of the wearable. (**Left**) The wearable is put in the wrist of the non-dominant hand. (**Right**) The electrodes are attached to the medial phalanges in the palm sides of index and middle fingers.

In our electronic design, guided by EDA features previously described, the EDA sensor measures DC exosomatic electrodermal activity through a couple of Ag/AgCl disc electrodes with contact diameters of 10 mm. The electrodes are attached to the medial phalanges in palm sides of index and middle fingers (see Figure 1). A small DC current is applied to the stratum corneum under the electrodes. Exceeding current must be limited to 10 μA/cm^2^ in order to avoid damage in the sweat gland ducts [24] and prevent nonlinearities in the current–voltage curve.

**Figure 2:**
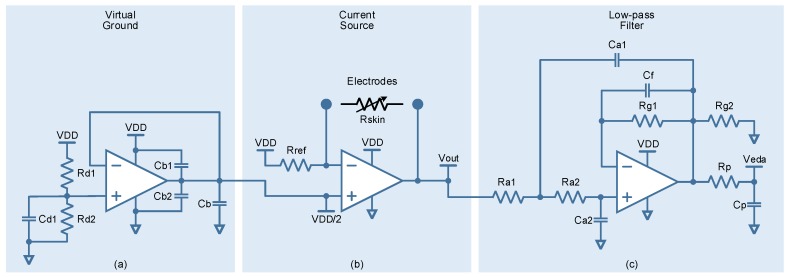
Electrodermal activity sensor building blocks. (**a**) Virtual ground. R${}_{d1}$ = R${}_{d2}$ = 100 kΩ, C${}_{d1}$ = 1 μF, C${}_{b1}$ = C${}_{b2}$ = 100 nF, and C${}_{b}$ = 47 μF; (**b**) Current source. R${}_{ref}$ = 825 kΩ; (**c**) Low-pass filter. R${}_{p}$ = 110 kΩ, C${}_{p}$ = 1 μF, R${}_{a1}$ = 75 kΩ, $R_{a2}$ = 150 kΩ, C${}_{a1}$ = C${}_{a2}$ = 1 μF, R${}_{g1}$ = R${}_{g2}$ = 10 kΩ, and C${}_{f}$ = 1 nF.

A single-supply, rail-to-rail input/output, precision operational amplifier, AD860x (Analog Devices) [25], implements a voltage-controlled linear current source (transconductor) as shown in Figure 2b. Such a compact solution is feasible, given that the load (stratum corneum) can be connected in a floating configuration, the input can provide all the required load current, and the current does not exceed the operational amplifier’s output current limit. In addition, a single-supply operation requires the generation of a virtual ground, usually at the halfway along power supply (V${}_{DD}$/2). Bearing this in mind, a resistor divider biasing technique (R${}_{d1}$ and R${}_{d2}$) has been used, buffered by a second operational amplifier, AD860x (Analog Devices), to provide a low-impedance ground at first operational amplifier’s non-inverting input, as shown in Figure 2a. C${}_{d1}$ forms a low-pass filter to eliminate conducted noise on the voltage rail. C${}_{b1}$ and C${}_{b2}$ are bypass capacitors and C${}_{b}$ is a bulk capacitor.

Therefore, the current fed into the skin is calculated as:

(1) $$I_{skin}=\frac{1}{2}\frac{V_{DD}}{R_{ref}}$$

In this regard, the R${}_{ref}$ = 825 kΩ reference resistor limits the current injected by the electrodes into the skin to a value around 2.546 μA/cm^2^, well below the 10 μA/cm^2^ recommendation.

Similarly, the skin resistance is calculated on the basis of the reference resistor as:

(2) $$R_{skin}=\left(1-\frac{2V_{out}}{V_{DD}}\right)R_{ref}$$

By taking the inverse of Equation (2), the skin conductance is obtained.

Finally, Figure 2c shows a third-order, low-pass filter with a cutoff frequency of about 1.5 Hz. The filter is implemented as a second-order low-pass active filter stage followed by a first-order passive RC (R${}_{p}$ and C${}_{p}$) low-pass filter stage. The active stage consists of a single-supply, low-pass Sallen-Key topology (R${}_{a1}$, R${}_{a2}$, C${}_{a1}$ and C${}_{a2}$) with a Butterworth response characteristic. The advantage of this second-order low-pass topology is that it only uses one operational amplifier, again AD860x (Analog Devices). Furthermore, this allows the addition of 2× gain (R${}_{g1}$ and R${}_{g2}$) to the signal chain. This filter limits the EDA signal bandwidth and accommodates its amplitude to the input dynamic range of the micro-controller’s analog-digital converter (ADC). C${}_{f}$ helps to the operational amplifier’s stability.

The sensor power rail is derived from a filtered 3.3 V system supply. Generous power supply bypassing and ground planes on the printed circuit board help to reduce noise.

## 3. Experimental Protocol

### 3.1. Experimental Design

Pictures from the International Affective Picture System (IAPS) have been chosen in order to trigger the desired arousal and valence levels [26], for the sake of eliciting distress and calmness. In fact, IAPS consists of a standard and categorized database of color photographs created to provide a wide range of affective stimuli. Moreover, the two primary dimensions recorded in the database are affective valence (ranging from pleasant to unpleasant) and arousal (ranging from calm to excited). So, for each IAPS picture the mean and standard deviation of arousal and valence is provided in four different tables constructed from the responses of men, women and children who responded by means of the Self-Assessment Manikin, an affective rating system [27], to the emotion felt when exposed to the pictures. According to the creators of the database, the existence of this image collection of normatively rated affective stimuli should: (1) allow better experimental control in the selection of emotional stimuli; (2) facilitate the comparison of results across several studies conducted in the same or different laboratory; and (3) encourage and allow exact replications within and across research labs who are assessing basic and applied problems in psychological science.

Thus, the idea is to use the IAPS database to show a series of images to some volunteer participants. Each image used in the experiment has to belong to one of the two classes, namely, high arousal-low valence and low arousal-high valence, corresponding to distress and calm, respectively, according to the circumplex affect model by Russell [28]. Obviously, high arousal-low valence does not directly mean “distress”, as “alarmed, tense, afraid, angry, annoyed and frustrated” are also classified in this quarter of the circumference. In the opposite low arousal-high valence side we have pleased, glad, serene, content, atease, satisfied, relaxed and calm. For this reason, the images taken as representative for the calm condition have a described arousal level lower than 4 and a valence level between 4 and 6. Similarly, the negatively distressed condition consists in samples with an arousal level higher than 5 and a valence level lower than 3.

The procedure for performing the experiment is described next. The participant sits in front of the experimentation monitor and the wearable is put in the wrist of the non-dominant hand (see Figure 1). In this regard, the experimentation monitor consists of a high resolution, 28 inch screen. When the technician verifies the proper functioning of the wearable and its communication with the software, the experiment starts. Firstly, the participant has to carefully read the general instructions of the experiment. Then, ten pictures that are labelled with high arousal and low valence are shown consecutively during 6 s each to the participant. Silences consisting of blank images with a fixed duration of 1 s are inserted before each picture used from the database.

The pictures are selected randomly from the set of images that fulfil the condition. Therefore, the segment used for subsequent analysis is 70 s long (10 pictures × 6 s duration, plus one blank image before each picture). In this sense, a single presentation of many stimuli presented for a short period of time might favour the continuity of emotional state [29]. Afterwards, a distracting task is presented to the participant so that his/her emotional state comes to neutral. Next, the experiment continues by showing randomly another set of ten images from IAPS that fulfil the condition to be part of those previously labelled as low arousal and high valence. Therefore, two segments of data from each individual are finally obtained, one for calm condition and another for distress condition. Again, silences are used before each picture. Lastly, the distracting task is offered again.

Thus, the total duration of the experiment for each participant is 140 s from the screening of the first image. The pauses are designed to allow the patient under study to recover from the previous stimulus. In this regard, the pictures are randomly shuffled, such that the order of viewing is different for each participant, albeit keeping the silence between two consecutive pictures. It is important to say that the experiments are carried out in the safest possible way. Accordingly, the participants are informed that they can stop visualizing the sequence in any moment. Moreover, the technician stays behind the participant during the whole experiment in order to assist at any time.

### 3.2. Study Population

Fifty participants (28 men and 22 women, mean age 23.54±2.64 years) have been enrolled in this experiment. All participants are informed about the high emotional contents of the pictures and they agree to be subjected themselves to the study. All participants are students from the Technical School at Cuenca, Spain. The scholars had to pass the PHQ-9 Depression Test Questionnaire to be accepted in the experiment. Unfortunately, four students were not approved, and one experiment was not valid due to technical problems. Thus, the number of valid experiments was forty-five (25 males and 20 females).

This study was carried out in accordance with the ethical standards of the responsible institutional committee on human experimentation. All subjects gave written informed consent in accordance with the Declaration of Helsinki.

## 4. Methodology

After the experimentation, all signals are segmented and processed according to the time duration of the stimuli. Next, the most significant time, morphological and frequency features are extracted. Finally, diverse statistical analysis and classification techniques are applied with the objective of maximizing the performance.

### 4.1. Signal Processing

The EDA raw signals are acquired from the wearable at a sampling rate of 10 Hz and a 12-bit resolution. These specifications are chosen to remain the EDA shape unaltered and to prevent distortions [19]. EDA signals result from the superposition of two different components, SCR and SCL. The sympathetic nervous system fires the sudomotor nerve, provoking the phasic response. Traditionally, the SCR intensity has been quantified after each elicited stimulus by detecting peaks directly on the EDA signal [19]. Next, the difference between a found peak and its preceding local minimum is assessed.

However, depending on the stimuli, it is frequent that SCRs appear as bursts, such that an EDA signal is represented as a sequence of consecutive SCRs. In this case, the SCR boundaries remain masked by the preceding response. Indeed, SCRs may occur at the rise or decay of existing stimuli, making it very difficult to determine if these responses correspond to a new stimulus or are part of previous events. In this regard, some works have defined different strategies for all possible overlapping cases [30]. Nevertheless, the through-to-peak standard method may result in an underestimation of the amplitude of consecutive SCRs, depending on the closeness among responses [31]. Recently, new studies have addressed this problem by decomposing the EDA signal into its two components by using a deconvolution operation [31]. Despite this approach requires more intensive signal processing, it has reported better performance than others that process directly the EDA raw data. Consequently, a similar methodology is applied in this work. Thus, considering the raw data from the wearable as y[n], a new 1.5 Hz cut-off low-pass FIR filter with order N=32 is applied over this signal in order to decrease the possible noise produced in the different electronic stages. The resulting filtered signal $\hat{y}\left[n\right]$ is calculated by means of a discrete convolution as:

(3) $$\hat{y}\left[n\right]=C_{0}y\left[n\right]+C_{1}y\left[n-1\right]+...+C_{N}y\left[n-N\right]=\sum_{i=0}^{N}C_{i}y\left[n-i\right]$$

Next, a deconvolution operation is carried out to separate SCR and SCL components. The deconvolution is an algorithm-based process used to reverse the effects of combining signals by finding the solution to the convolution equation, such that:

(4) $$\hat{y}\left[n\right]=\left(r\times l\right)\left[n\right]=\sum_{i=0}^{N}r\left[n-i\right]l\left[i\right]$$

being × the convolution operator in the time domain, $\hat{y}\left[n\right]$ the filtered EDA signal, r[n] the required SCR and l[n] the SCL components. It is worth noting that l[n] corresponds to the transfer function in Equation (4), such that, if l[n] is known or estimated, a deterministic deconvolution could be used to compute the desired component r[n].

**Figure 3:**
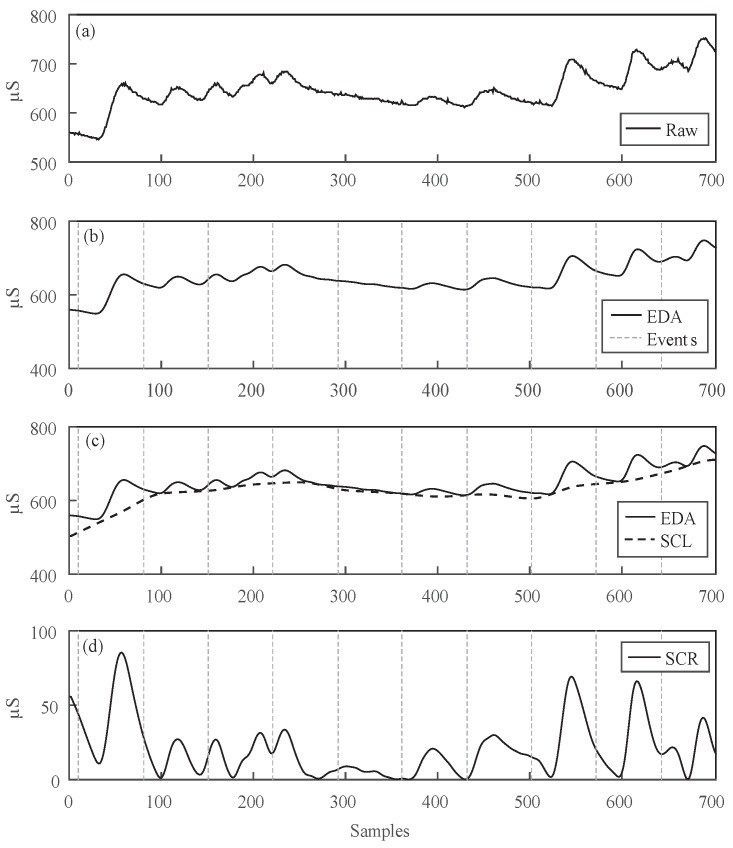
Different stages in EDA signal processing. (**a**) Raw EDA signal before filtering. (**b**) Raw EDA signal after low-pass filtering and stimuli onset. (**c**) Estimation of EDA baseline using a cubic spline approximation. (**d**) Skin conductivity response (SCR) obtained after the convolution process.

In this regard, three different assumptions have been considered in this work. Firstly, the exact moments when the stimulus (picture) starts have been recorded as events, as can be observed in Figure 3b. Secondly, notice that the SCR component takes a while since the stimulus is fired until the sympathetic system reacts through filling the sweat glands, thus increasing the skin conductivity. Indeed, the exact time occurrence of SCR varies depending on skin type and genetic aspects [22]. Furthermore, the SCR duration varies subject to the stimulus’ nature and the participant’s reaction against such stimulus. Consequently, a fixed temporal window (5 s) is used as the time segment where the SCR response may occur, following the recommendations of a previous work [17]. Thus, a period from +1 to +6 s after the onset of each stimulus is considered. In third place, l[n] corresponds to $\hat{y}\left[n\right]$ when no stimulus is elicited [19].

Considering the aforementioned assumptions, the time intervals occurring before and after each phasic impulse are used to estimate the SCL gaps between the different phasic impulses. In this work, a cubic spline fit is used to approximate l[n] at the gaps produced in the SCR temporal window, as you may observe in Figure 3c. Once l[n] is known, r[n] can be computed by following the inverse process defined in Equation (4). Nevertheless, given the complexity of this operation in time, it is preferable to work in the frequency domain, where convolution and deconvolution become in simple multiplications and divisions. Thus, $\hat{y}\left[n\right]$ and l[n] are transformed into the frequency domain by using the Fourier Fast Transforms (FFT), such that r[n] can be calculated as:

(5) $$r{\left[n\right]}_{F}=\frac{\hat{y}{\left[n\right]}_{F}}{l{\left[n\right]}_{F}}$$

being $\hat{y}{\left[n\right]}_{F}$, $r{\left[n\right]}_{F}$ and $l{\left[n\right]}_{F}$ the FFTs of EDA signal, and SCR and SCL components, respectively. The original r[n] component alone is estimated by computing the inverse Fourier transform over $r{\left[n\right]}_{F}$. As it can be observed in Figure 3d, r[n] corresponds to a signal with zero baseline, where each impulse reflects the activation of the sudomotor nerve.

### 4.2. Feature Sets

In the present section, all the features are estimated. The characteristics related to time domain, frequency domain, statistics and morphological analysis are computed for each physiological variable. In this work, thirty six features are used, as you may observe in Table 1.

Different time-domain and frequency-domain markers are computed over the phasic component SCR to characterize the EDA signal. The SCL tonic component is out of scope of this study, since it uses to vary among different people due to physical and genetic aspects [19]. Firstly, a number of temporal metrics are computed over the SCR component. Thus, the mean (*MSC*), standard deviation (*SDSC*), maximum (*MASC*), minimum (*MISC*) and the dynamic range (*DRSC*), defined as the difference between *MASC* and *MISC*, are estimated. In order to highlight the sudden changes in the skin conductivity, the first and second derivative of SCR are also computed. Then, the mean (*FMSC*) and standard deviation (*FDSC*) of the first derivative and the mean (*SMSC*) and standard deviation (*SDSC*) of the second derivative are calculated.

In addition, several morphological features are defined for SCR characterization. Thus, in order to identify the morphological alterations produced when SCRs are present in the EDA signal, the arc length (*ALSC*) is computed as:

(6) $$ALSC=\sum_{n=2}^{N}\sqrt{1+{\left(r\left[n\right]-r\left[n-1\right]\right)}^{2}}.$$

This parameter discerns between the presence or absence of SCR components, and it has been used previously in the morphological characterization of Gaussian responses [32]. Moreover, some parameters related to the SCR amplitude are also used to assess the activation of the sympathetic nervous system. Thus, the integral (*INSC*), normalized average power (*APSC*) and normalized root mean square (*RMSC*) of SCR are calculated as:

(7) $$INSC=\sum_{n=1}^{N}\left|r\left[n\right]\right|,$$

(8) $$APSC=\frac{1}{N}\sum_{n=1}^{N}r{\left[n\right]}^{2},$$

(9) $$RMSC=\sqrt{\frac{1}{N}\sum_{n=1}^{N}r{\left[n\right]}^{2}}.$$

Furthermore, possible relationships between the amplitude and energy of the SCR signal with its arc length are studied. Thus, the area-perimeter (*ILSC*) and the energy-perimeter (*ELSC*) relationships are estimated as the ratio between *INSC* and *RMSC* with *ALSC*, respectively. Finally, high order skewness (*SKSC*) and kurtosis (*KUSC*) statistics, as well as the central moment (*MOSC*) are calculated on the SCR component. These metrics assess the symmetry and shape of a probability distribution, and can therefore be also considered as geometrical descriptors. Indeed, if an SCR is considered as a Gaussian distribution, *SKSC* indicates if the tail distribution is longer at the left or at the right relative to the normal distribution. Similarly, the *SKSC* shows the degree of peakedness or flatness of a distribution relative to the normal distribution.

In regards to frequency aspects, the SCR component is transformed into the frequency domain by using a nonparametric FFT algorithm. Then, the spectral power in bandwidths 0.1 to 0.2 (*F1SC*), 0.2 to 0.3 (*F2SC*) and 0.3 to 0.4 (*F3SC*) Hz are estimated. These bands have been previously used in other studies [33].

### 4.3. Statistical Analysis

Shaphiro-Wilks and Levene tests have proved that the distributions are normal and homoscedastic for all the features studied. Consequently, the results are expressed in terms of the mean ± standard deviation for all the samples belonging to a same class. The statistical differences between both classes, calm and distress, are assessed by a one-way ANOVA test. A value of statistical significance ρ< 0.05 has been considered as significant.

Moreover, a 10-fold stratified cross-validation is used to assess the discriminant ability of each feature. This kind of cross-validation allows to obtain a highly reliable performance generalization of the metric under study [34]. Indeed, this approach makes use of all available data both for training and testing. This avoids the possibility of the classification results to be highly dependent on the choice of a given training-test segmentation. Thus, the database is firstly partitioned into 10 equally sized folds, rearranging the data to ensure that each fold is a good representative of the whole. Then, 10 training and validation iterations are performed, such that a fold of the data is held out for test, whereas the other ones are used for learning within each iteration. For each learning set, a receiver operating characteristic (ROC) curve is used to obtain the optimal discriminant threshold between calm and distress condition. The ROC curve is created by plotting the true positive (TP) rate against the false positive (FP) rate at various threshold settings. Here, the TP rate (or sensitivity) is considered as the percentage of distress condition correctly classified. On the other hand, the FP rate (or one-specificity) corresponds to the rate of calm individuals improperly identified. The optimal threshold is selected as the value which provides the highest accuracy. Finally, the global precision is obtained by averaging this procedure 10 times.

Additionally, the relationships among the different temporal, morphological and frequency features are analysed by means of decision trees. In brief, the algorithm starts taking into account all the input data, examining all the possible splits on each feature. Then, the split with the best optimization criterion, based on the Gini diversity index [35], is chosen. In this respect, the Gini index is commonly computed by using the Lorenz curve, and it is expressed mathematically as:

(10) $$G=\frac{a}{a+b}$$

where *G* corresponds to the Gini index, *a* represents the area that lies between the line of equality and the Lorenz curve and *b* represents the area under the Lorenz curve. Thus, a node containing observations from just one group (pure node) has Gini index 0, while if the node contains observations from both groups (impure node), the Gini index is a positive number, ranging between 0 and 1. Finally, when the split has been executed, the aforementioned process is repeated recursively for the two child nodes using the remaining data. It is worth noting that some stopping rules are imposed to prevent tree overgrowth. Thus, the growth of every tree is always stopped when any node only contains samples from a group of subjects (pure node) or less than 20% of all samples.

## 5. Results

Table 2 shows the mean and standard deviation of the features calculated. Only those markers reporting statistical differences throughout a one-way ANOVA test are presented.

Concerning the study of the SCR component, 16 out of 22 features report significant differences. In this respect, higher values of skin conductivity are observed in the distress class regarding the calm class for all temporal parameters. From a statistical point of view, all temporal parameters achieve a comparable power discrimination between both classes. On the other hand, a number of morphological markers are defined and used in this analysis. Most of them show an important discriminatory power. It is worth noting that ILSC achieves the most remarkable trends, while ALSC reports the lowest significance. Just like in the temporal parameters, the average values of the morphological markers show an increasing pattern, such that higher skin conductivities are observed in distress compared to calm condition. Regarding the frequency parameters, all the markers report a power increase when the subjects are elicited with stressing stimuli. In this regard, F2SC experiences the highest difference between the classes, achieving a considerable statistical significance.

In order to estimate a reliable and robust classification accuracy for each studied parameter, the stratified 10-fold cross-validation is run five times. Thus, the average values of sensitivity, specificity and accuracy, reported by each marker and for both training and test subsets iterations, are shown in Table 3.

The classification results are in agreement with the discriminatory power obtained previously. Thus, all the temporal parameters computed over SCR component achieve a similar global accuracy, ranging from 71% to 73%. Similarly, morphological parameters computed over SCR component reach similar performance with global accuracy varying between 73% and 75%. Nevertheless, the energetic parameters *APSC* and *RMSC* show a very notable classification accuracy, reaching a correctness of 79.28% and 79.94%, respectively. In agreement with the discriminatory power shown before, the arc length (*ALSC*) achieves the worst performance in the classification. On the contrary, the area-perimeter marker *ILSC* reports the highest global accuracy among the morphological features, achieving a precision of 79.99%. Finally, frequency markers show a very notable performance, where *F2SC* reaches the highest global accuracy among all the studied parameters. It is important to remark that almost all markers show a good balance between sensitivity and specificity. Nevertheless, some remarkable parameters state a slight increase in sensibility, i.e. an increase in the ability of detecting distress condition. Hence, *RMSC*, *ILSC*, *F2SC* and *F3SC* report sensibility values higher than 80%.

Considering this context, a series of tree-based classification models are programmed in order to study the possible relationships among the different parameters. Here we present the tree-based discriminant model that reaches the highest performance. This model is constructed by exclusively considering temporal and morphological features. In this case, the frequency parameters are excluded, thinking in a lighter algorithmic model only focused in analyzing the time domain. In this model, a threshold value of 4.4830 on *SDSC* parameter in this model serves to divide into two subgroups. Then, one of the subgroups is assessed by means of *SKSC* parameter, taking value 3.1102 as classification threshold. This tree-based model achieves a sensitivity, specificity and accuracy of 93.9%, 85.36% and 89.18%, respectively. In this regard, the model improves the global correctness more than 8%, regarding the performance of the best single parameter *F2SC*. It is also important to highlight that sensitivity, that is, the ability to discriminate distress condition, improves over 9%.

## 6. Conclusions

A recent paper [36] says that “Stress is a very complex subject and measuring stress is not an easy task. There are many markers that could be used, many algorithms that could be applied, and many forms of stress which could be observed”. Moreover, an important number of works can be found in the literature based in distress detection. Although comparison among works should always be considered with caution, since different ways to provoke the stress may trigger several cognitive processes [22], it is worth noting that the present study has achieved a comparable performance to other systems aimed at classifying calm and distress emotional states. Thus, while this work has reported a global accuracy around 89% when classifying calm and distress condition, other works based on electrodermal activity as reported in very interesting reviews on wearable sensors for remote health monitoring [37,38,39], state stress detection rates ranging from 75% to 95% [40,41,42,43]. Nevertheless, complex classifiers have been used to improve their performance.For instance, an approach throws a precision of 75% using EDA after classifying with k-means, Gaussian mixture models, SVM and decision trees [44].

On the other hand, it is important to highlight that these and/or other approaches have used an important number of features from more than one physiological sensor to calculate the stress degree. For instance, electroencephalogram, electrocardiogram and facial cameras complement EDA signals to reach an accuracy of 68% [45]. The classification is performed by means of support vector machines (SVM) and naive Bayes classifier. In another approach [46], heart rate variability (HRV) supplements EDA to obtain 78% of precision, also using SVM classifiers. Two other papers using EDA and HRV reach an accuracy of 97.3% [10] and 80% [47], respectively. However, in the first article [10] a driving task is analysed with a series of sensors: hand and foot EDA, together with three other physiological measurements, namely HRV, Trapezius muscle electromyography and respiration. Moreover, the other work [47] uses EDA, blood pressure, HRV, eye gaze and pupil dilation. Another work with three physiological measurements [48], which are HRV, skin temperature and EDA, gets a precision of 91.2%. In another work [49], a portable embedded device to measure the accumulated stress level is designed. This device uses EDA and HRV with electrocardiograph or photoplethysmograph signals to obtain an accurate stress level.

On the contrary, our proposal uses only features from SRC to achieve a high performance that is comparable to the most remarkable works. In this sense, the most outstanding aspect of our contribution is the development of the necessary hardware, signal processing and classification model to deploy a wearable device with a high ability to discriminate between the two considered states. The simplicity of the classification model and the lightness of the signal processing approach enables this device to work in real-time and long-term. Another relevant aspect is that almost all the features computed on the SCR component show some degree of relevance in the classification.

For the sake of comparability and reproducibility of the experiment described in this study, a DC-EXM method has been chosen for the acquisition of EDA signals. Indeed, most of the works using EDA, and found in scientific literature, have been performed by means of this methodology [19]. Although ESM approaches may provide some advantages over the EXM methods, as, for instance, no need of additional amplification and coupling circuitry, the output signal generated is biphasic or even triphasic, which difficult considerably the interpretation and further processing of the data [19]. From a point of view of electronic design, many efforts have been made to standardize the use of constant voltage versus constant current sources when using DC-EXM methodology. Despite constant voltage directly providing conductance values, there is no consensus in the literature about its generalized use, because the conductance value can be obtained easily with constant current sources if SCL is also measured. In this aspect, the design of constant current sources are preferred because they are easier to design and present less tolerance than constant voltage variants.

Finally, some limitations should be considered. First of all, the wearable prototype has been tested in laboratory conditions enrolling exclusively young subjects. Therefore, the results of this work can not be generalized directly to the entire population. With the aim of validating the results obtained, people of all ages will be enrolled in future studies. Furthermore, a sequence of 10 consecutive pictures with lengths of 6 s have been used as stimuli in this work. Considering such duration for visualization, the cognitive dimension of exploration of images during the 6 s may affect the outcomes. In this line, there is no consensus about which type of stimulus (image, sound, or video clip) and duration is the most adequate to elicit certain emotional states [22]. Moreover, it should be noted that the same order of condition high arousal-low valence—low arousal-high valence could affect, which could suppose an experimental bias.

## Figures and Tables

**Table 1:** Temporal, morphological and frequency features computed for EDA signals.

Analysis	Features
Temporal	*MSC, SDSC, MASC, MISC, DRSC, FMSC, FDSC, SMSC, SDSC*
Morphological	*ALSC, INSC, APSC, RMSC, ILSC, ELSC, KUSC, SKSC, KUSC, MOSC*
Frequency	*F1SC, F2SC, F3SC*

**Table 2:** Results obtained from skin conductance response (SCR). Mean and standard deviation values for emotional states of calm and distress, and statistical significance (ρ), for all parameters are presented.

Feature	Calm Condition	Distress Condition	ρ
**Acronym**	**Mean ± Std**	**Mean ± Std**	
*MSC*	5.5339 ± 4.2228	13.0193 ± 8.6201	1.03 $\times \;10^{-5}$
*SDSC*	4.4618 ± 4.8976	12.5249 ± 9.0340	1.33 $\times \;10^{-5}$
*MASC*	28.6079 ± 27.44	69.4104 ± 48.0310	2.68 $\times \;10^{-5}$
*DRSC*	28.5653 ± 27.4660	69.3719 ± 48.0145	2.67 $\times \;10^{-5}$
*FDSC*	0.9932 ± 0.9665	2.2660 ± 1.6756	1.50 $\times \;10^{-4}$
*ALSC*	1.4049 $\times 10^{4}$ ± 99.6809	1.4153 $\times \;10^{4}$ ± 279.7989	0.0175
*INSC*	193.9833 ± 148.3517	457.2628 ± 304.9061	1.11 $\times \;10^{-5}$
*APSC*	4.6324 ± 9.2181	23.8873 ± 36.4345	0.0026
*RMSC*	7.3106 ± 6.3476	18.0970 ± 12.4265	1.20 $\times \;10^{-5}$
*ILSC*	5.5067 ± 4.1480	12.8120 ± 8.2006	7.31 $\times \;10^{-6}$
*ELSC*	0.0065 ± 0.0129	0.0330 ± 0.0484	0.002
*SKSC*	1.8838 ± 1.1882	3.1146 ± 0.7159	0.0031
*MOSC*	2.2337 ± 5.0694	11.7973 ± 19.7930	0.0057
*F1SC*	2.9219 ± 5.4380	14.1513 ± 20.2989	0.0018
*F2SC*	0.1631 ± 0.2984	1.4143 ± 2.1767	8.99 $\times \;10^{-4}$
*F3SC*	0.1391 ± 0.3231	1.2288 ± 2.3907	0.0076

**Table 3:** Sensitivity (Se), specificity (Sp) and accuracy (Ac) for all the parameters under study and for training and test subsets.

Feature	Learning			Test		
**Acronym**	**Se (%)**	**Sp (%)**	**Ac (%)**	**Se (%)**	**Sp (%)**	**Ac (%)**
*MSC*	75.95	83.10	79.52	69.00	76.78	72.95
*SDSC*	85.06	78.95	82.43	78.57	76.07	77.38
*MASC*	81.45	76.35	78.91	74.07	72.28	73.34
*DRSC*	82.92	75.42	79.18	76.64	71.00	73.62
*FDSC*	74.58	80.43	77.50	69.21	74.85	71.81
*ALSC*	72.15	76.86	74.52	70.92	75.64	73.19
*APSC*	84.98	79.88	82.43	79.92	78.71	79.28
*RMSC*	85.92	78.92	82.42	81.57	78.85	79.94
*ILSC*	85.12	79.71	82.42	81.78	78.14	79.99
*ELSC*	71.78	73.14	72.25	63.00	75.35	69.16
*SKSC*	77.44	81.08	79.29	73.28	75.71	74.53
*MOSC*	86.19	78.75	82.50	75.28	75.21	75.18
*F1SC*	77.15	77.94	77.57	74.85	75.85	75.38
*F2SC*	85.29	85.93	85.61	84.92	78.50	81.61
*F3SC*	85.12	79.71	82.42	80.35	76.42	78.30

## Abbreviations

The following abbreviations are used in this manuscript:

EDA	electrodermal activity
IAPS	International Affective Picture System
SCL	skin conductance level
SCR	skin conductance response
SMNA	sudomotor nerve activity
